# Prevalence of Frey syndrome following extraoral surgical treatment for mandibular fractures: a systematic review and meta-analysis

**DOI:** 10.12688/f1000research.140994.1

**Published:** 2023-09-14

**Authors:** Evangelos Kostares, Michael Kostares, Georgia Kostare, Maria Kantzanou

**Affiliations:** 1 Microbiology, National and Kapodistrian University of Athens, Athens, Attica, 115 27, Greece; 2 National and Kapodistrian University of Athens, Athens, Attica, 115 27, Greece

**Keywords:** Frey syndrome;mandibular fractures;open reduction and internal fixation;ORIF;prevalence;meta-analysis

## Abstract

Our study aims to estimate the prevalence of Frey syndrome following open reduction and internal fixation (ORIF) for mandibular fractures. Two reviewers independently conducted a systematic literature search in the Medline and Scopus databases. The pooled prevalence with 95% confidence intervals (CI) was estimated, and quality assessment, outlier analysis, and influential analysis were performed. In total, fifteen eligible studies were included in this meta-analysis. One study was identified as critically influential. The overall prevalence of Frey syndrome following extraoral surgical treatment for mandibular fractures was estimated as 0.01% (95%CI 0%-0.7%) with moderate heterogeneity observed between studies. In the meta-regression analysis with continuous variables, no statistically significant association was observed. Despite the relatively low prevalence, the impact of Frey syndrome on affected individuals should not be underestimated. Additional research will provide a more comprehensive understanding of the underlying factors contributing to Frey syndrome, leading to improved preventive measures and treatment strategies. A better grasp of the prevalence and associated risk factors will aid in the development of guidelines to minimize the occurrence of this syndrome.

## Introduction

Mandibular fractures, a prevalent form of facial trauma, often necessitate surgical intervention. ^1^ Such fractures can result from a range of causes ^2^^,^^3^ and are more frequently observed in middle-aged males. ^4^ The condyle is the most commonly affected site, constituting a significant proportion of all mandibular fractures. In many instances, these fractures can lead to life-threatening complications like airway blockage and significant bleeding. ^5^ Consequently, it is crucial to promptly identify and treat such cases. Commonly, the management of mandibular fractures is conducted within the confines of hospital facilities, typically undertaken by surgical specialists with relevant expertise in this domain ( *e.g.*, oral and maxillofacial surgeons (OMFS), torhinolaryngologists or other medical specialities). Treatment approaches encompass closed reduction or surgical intervention via intraoral or extraoral methods. ^2^^,^^3^^,^^5^^,^^6^ Despite being widely esteemed as an efficacious and secure procedure, open reduction and internal fixation (ORIF) introduces significant intricacies, prominently including inferior alveolar nerve (IAN) impairment, surgical site infection (SSI), exposure of osteosynthesis material, temporomandibular joint issues, risk for hemorrhage, Frey syndrome, soft or hard tissue necrosis, malocclusion, and other complications. ^7^^‐^^11^

Frey syndrome (which is named after Łucja Frey) arises from an unusual reinnervation process following damage to the auriculotemporal nerve. This nerve, a branch originating from the trigeminal nerve, consists of parasympathetic fibers that regulate saliva production in the parotid gland, as well as sympathetic fibers that control the sweat glands on the face and scalp. Injuries resulting from surgical interventions such as, parotidectomy, facelifts, ORIF for mandibular fractures, as well as infections and traumas, can lead to damage in the parasympathetic and sympathetic nerve fibers of the auriculotemporal nerve within the parotid region. This, in turn, triggers an aberrant regrowth of post-ganglionic parasympathetic nerve fibers responsible for the production of saliva. These regenerated fibers deviate from their normal course and follow the existing sympathetic pathways towards the blood vessels and sweat glands of the skin, ultimately leading to the onset of Frey syndrome. Individuals afflicted with Frey syndrome commonly exhibit indications such as facial warmth, flushing, and perspiration, particularly occurring during meals, within the region influenced by the auriculotemporal nerve. This encompassed area consists of the skin, the ear, the temporal region, the scalp, and the temporomandibular joint. ^12^^,^^13^ Frey syndrome diagnosis relies on patient history (subjective approach), while verification can be achieved using objective techniques like the Minor starch-iodine test or infrared thermography. The starch-iodine test involves applying iodine to the surgically affected area, followed by the application of dry starch after the iodine dries. Upon introducing a salivary stimulus, the presence of iodine and sweat causes the starch to change color to blue/brown. Thermography is a method for diagnosing Frey's syndrome with a quantitative thermal difference between operated and unoperated facial regions ^13^^‐^^15^

The prevalence of Frey syndrome subsequent to extraoral surgical treatment for mandibular fractures exhibits significant heterogeneity across scientific publications. Consequently, the primary aim of the present investigation is to provide a more accurate assessment of the occurrence of Frey syndrome following ORIF, through a meta-analysis of the existing data found in the scientific literature.

## Methods

### Search strategy

**Figure 1.**
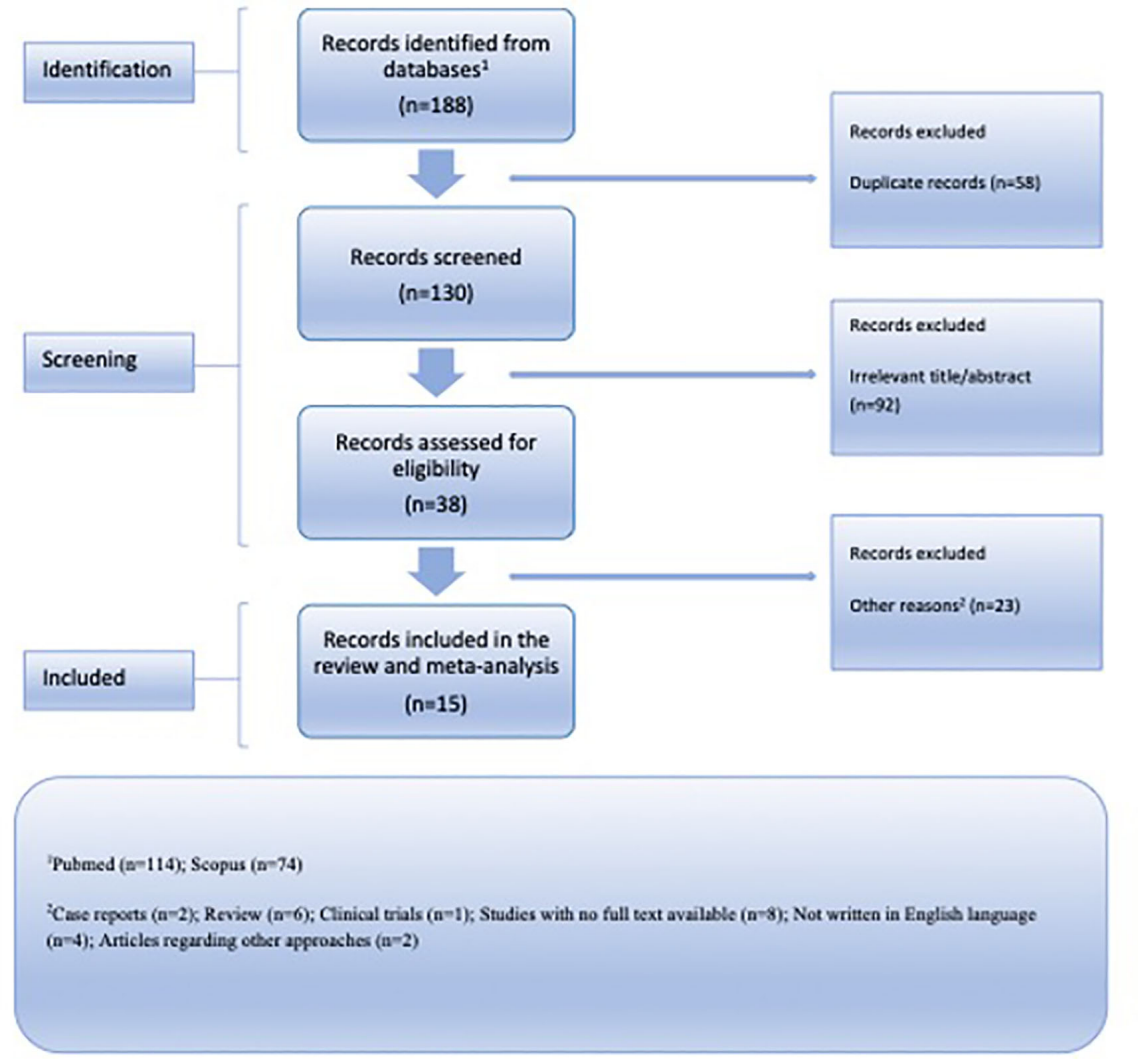
Flow chart illustrating the organized presentation of outcomes derived from the identification and choice of pertinent research studies.

The Medline (PubMed search engine) and Scopus database searches were conducted in a thorough manner, adhering to the guidelines set by the Preferred Reporting Items for Systematic Reviews and Meta-Analysis (PRISMA), ^16^ ensuring a meticulous and rigorous approach as depicted in Figure 1. The PRISMA checklist was utilized to facilitate the systematic review process. Articles published up until May 1st, 2023, were collected. An independent literature search was conducted by two reviewers using the following keywords: “ *mandibular*”, “ *mandible*”, “ *jaw*”, “ *fractures*”, “ *open reduction and internal fixation*”, “ *ORIF*”, “ *surgical treatment*”, “ *Frey syndrome*”, “ *Baillarger's syndrome*”, “ *gustatory hyperhidrosis*”, “ *auriculotemporal syndrome*”, “ *Dupuy syndrome*”, “ *prevalence*”, “ *incidence*”, “ *rate*”. In conjunction with the primary search, a thorough examination of the reference lists from the identified studies was conducted to identify any additional articles that may have been overlooked. The collected studies were meticulously organized and stored utilizing the Zotero reference management software (version 6.0.26). ^17^ The credibility of our dataset was ensured by diligently removing any duplicate references. Following the initial search, the remaining articles were thoroughly examined by two independent investigators. The study selection process comprised two distinct stages. Initially, the titles and abstracts of the articles were meticulously reviewed, leading to the elimination of those not meeting the predetermined inclusion criteria. In the second stage, the full texts of the remaining articles were obtained and subjected to a comprehensive evaluation. Any disagreements during the study selection were resolved through consensus among the team members, ensuring a consistent and unified decision-making process. By employing this systematic approach, a comprehensive and dependable collection of studies for analysis was achieved.

### Criteria for study selection and data extraction

Articles that specifically investigated the prevalence of Frey syndrome following surgical interventions through extraoral incision for mandibular fractures were included with no restriction on publication date. Excluded from consideration were case reports, case series involving fewer than five participants, review papers, systematic reviews, meta-analysis, randomized or non-randomized clinical trials, studies involving animals, studies lacking complete text, articles not in English language, studies regarding other approaches (intraoral), ^18^ articles examining Frey syndrome per fracture and articles containing data derived from surveillance databases. The subsequent factors were extracted from each study: the name of the primary author, publication year, research design, geographical region of origin, country of origin, study duration, overall number of patients, percentage of male participants, mean age, and individuals experiencing postoperative Frey syndrome.

### Quality assessment

To evaluate the quality of the studies included, two investigators independently assessed them using the National Heart, Lung, and Blood Institute (NHLBI) Quality Assessment tool for Observational Cohort and Cross-Sectional Studies. The evaluation process entailed a thorough examination of each study to identify any methodological or survey implementation weaknesses that could impact internal validity. During the assessment, the investigators considered 14 specific questions to gauge the quality of each study. They were provided with response options such as “yes,” “no,” “cannot determine” ( *e.g.*, in instances where the data presented uncertainties or contradictions), “not reported” ( *e.g.*, in cases where data were not reported or were incomplete), or “not applicable” ( *e.g.*, when a question did not pertain to the specific type of study under evaluation). By evaluating these questions, the investigators categorized the risk of bias for each study as either “low,” “moderate,” or “high,” enabling an overall assessment of the study's quality. ^19^ By conducting this rigorous quality appraisal, our aim was to ensure that only studies demonstrating a moderate or high level of internal validity were included in our analysis.

### Statistical analysis

Using RStudio software (version: 2022.12.0+353) by RStudio Team (2022), ^20^ a meta-analysis was executed. The meta-analysis, facilitated by the metafor package, ^21^ involved estimating the pooled prevalence and its associated 95% confidence intervals (CI) using the DerSimonian and Laird random-effects model, complemented by a Freeman-Tukey double arcsine transformation. ^22^ The existence of heterogeneity among studies was assessed by visually examining the forest plot and employing Cochran's Q statistic along with its corresponding p-value. The Higgins I ^2^ statistic, accompanied by its 95% CI, was employed to quantify the extent of genuine heterogeneity in effect sizes. An I ^2^ value falling within the ranges of 0%-40%, 30%-60%, 50%-90%, and 75%-100% denoted insignificant, moderate, substantial and considerable heterogeneity, respectively. To determine if the potential outlying effect sizes were also influential, screening for externally studentized residuals with z-values larger than two in absolute value and leave-one-out diagnostics were performed. ^23^ Due to a paucity of data regarding categorical variables, such as surgeon’s level, subgroup analysis was not performed. In the conducted meta-regression analysis involving continuous variables, an investigation was carried out to assess the influence of publication year and the proportion of male participants as moderators impacting the relevant effect sizes. Due to the limited availability of data on additional continuous variables like the mean age of the patients, the analysis was performed using the aforementioned ones. ^24^ Unless otherwise stipulated, the statistical significance was established at p = 0.05 (two-tailed). Methods designed to assess publication bias, including Egger’s test ^25^ and Begg’s test ^26^ along with the utilization of funnel plots, were originally formulated within the framework of comparative data. These methods operate under the assumption that studies with positive outcomes are more likely to be published than those with negative outcomes. Nevertheless, in a meta-analysis centered on proportions, the criteria for defining a positive result lacks clarity and consensus. ^27^ Consequently, in this current meta-analysis, an evaluative approach was adopted to qualitatively assess potential publication bias.

## Results

### Results and characteristics of the included studies

In aggregate, this analysis encompassed a grand total of 15 studies, collectively involving a cohort of 622 participants. These publications spanned the period from 2000 to 2020, encapsulating research carried out between 1990 and 2019. Among these studies, one adopted a cohort design, ^42^ while the remaining were cross-sectional in nature. ^28^^‐^^41^ Geographically, the studies were primarily executed across Asia (including India, Japan, Turkey, and Nepal), followed by Europe (including Italy, France, Austria, and Switzerland), and finally America (including the USA and Canada). The representation of male participants averaged at 79.9%, and the mean age of participants ranged from 27 to 41.5 years, with a median age of 29.2 years. The assessment of study quality consistently categorized all the studies as being of moderate quality. Their detailed characteristics are outlined in Table 1.

**Table 1.** Descriptive characteristics of the included studies.

Author	Year of publication	Study design	Continent of origin	Country	Study period	Total patients	Proportion of males (%)	Mean age (years)	Frey syndrome	Quality assessment
Ellis E. ^28^	2000	cross-sectional	America	USA	1990-1997	93	81.7	NA	0	Moderate
Narayanan V. ^29^	2009	cross-sectional	Asia	India	NA	31	NA	NA	0	Moderate
Croce A. ^30^	2010	cross-sectional	Europe	Italy	NA	13	84.6	33	1	Moderate
Benech A. ^31^	2011	cross-sectional	Europe	Italy	2006-2008	14	71.4	33	0	Moderate
Bindra S. ^32^	2011	cross-sectional	Asia	India	NA	10	100	31.6	0	Moderate
Ebenezer V. ^33^	2011	cross-sectional	Asia	India	2008-2010	20	NA	NA	0	Moderate
Rao J.K.D. ^34^	2013	cross-sectional	Asia	India	NA	15	100	NA	0	Moderate
Yabe T. ^35^	2013	cross-sectional	Asia	Japan	1997-2012	14	71.4	28.6	0	Moderate
Bouchard C. ^36^	2014	cross-sectional	America	Canada	2003-2012	108	75	35.6	1	Moderate
Spinzia A. ^37^	2014	cross-sectional	Europe	Italy	2003-2011	25	72	27	2	Moderate
Zrounba H. ^38^	2014	cross-sectional	Europe	France	2005-2010	141	75	35	1	Moderate
Aslan C. ^39^	2016	cross-sectional	Asia	Turkey	2012-2014	24	66.7	34.6	0	Moderate
Pau M. ^40^	2016	cross-sectional	Europe	Austria	2012-2015	42	76.2	NA	0	Moderate
Bruneau S. ^41^	2017	cross-sectional	Europe	Switzerland	2007-2015	43	86	41.5	0	Moderate
Koirala U. ^42^	2020	cohort	Asia	Nepal	2016-2019	29	79.3	29.8	0	Moderate

NA: not applicable.

### Prevalence of Frey syndrome following extraoral surgical treatment for mandibular fractures

**Figure 2.**
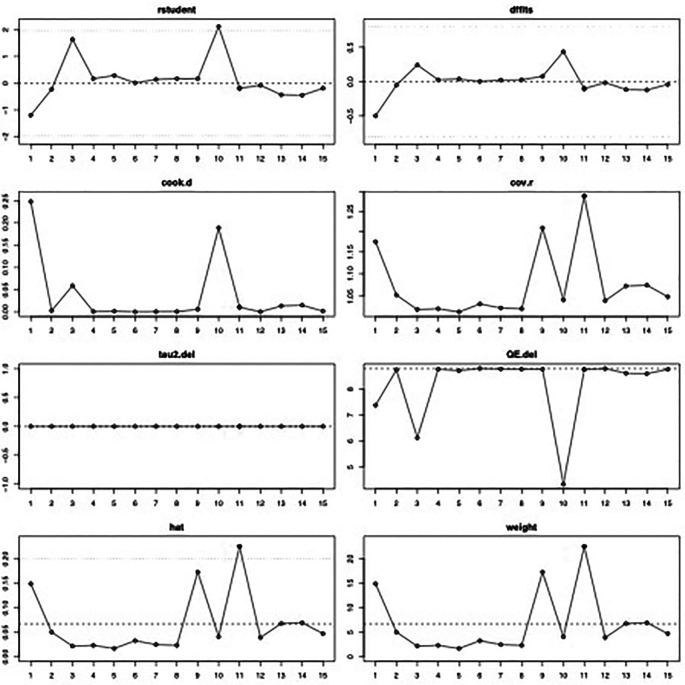
Visual representation of the influence diagnostics for each of the included studies regarding the prevalence of Frey syndrome after ORIF for mandibular fractures.

**Figure 3.**
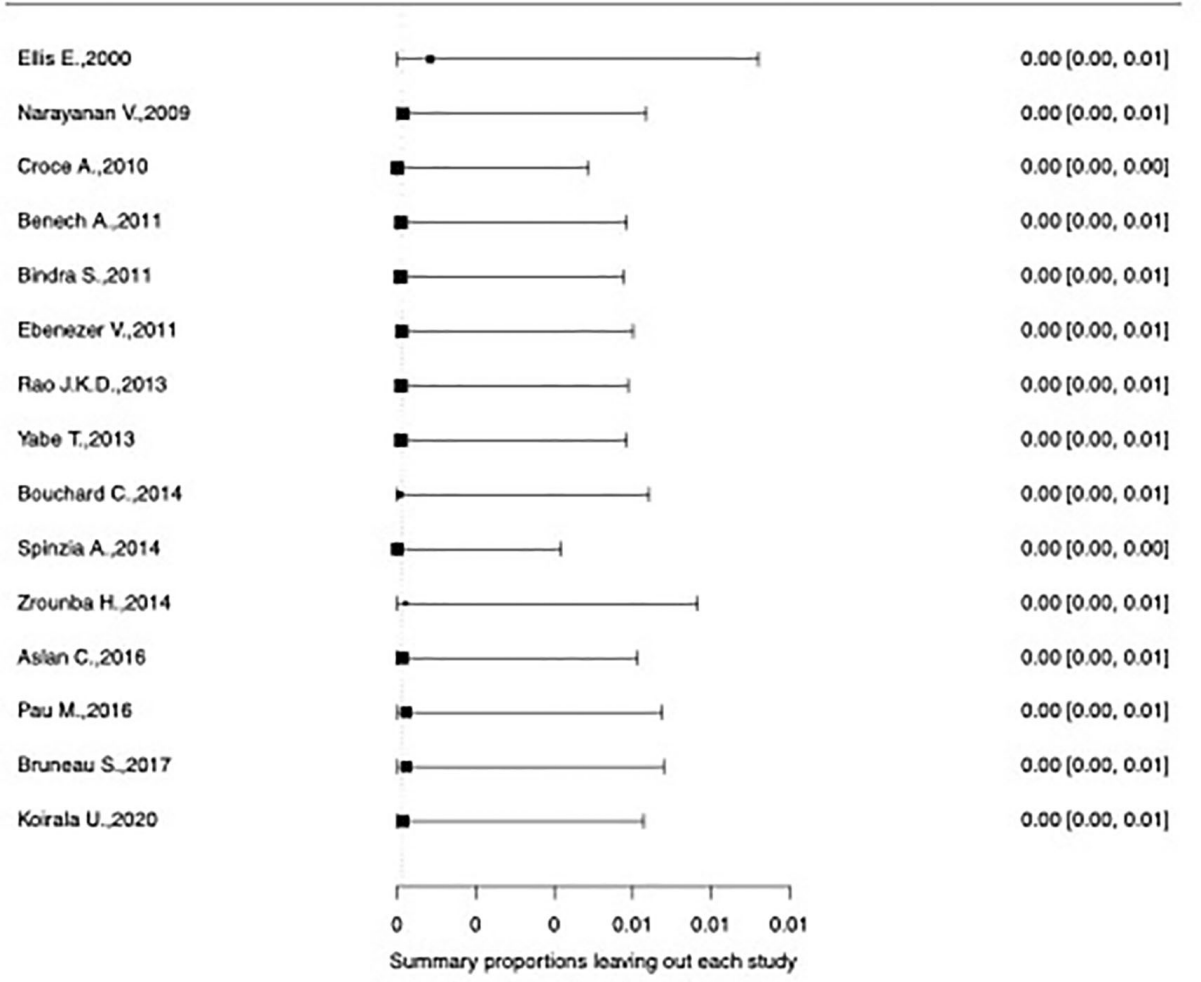
Forest plot displaying the re-calculated pooled effects, with one study omitted each time, using the leave-one-out method.

**Figure 4.**
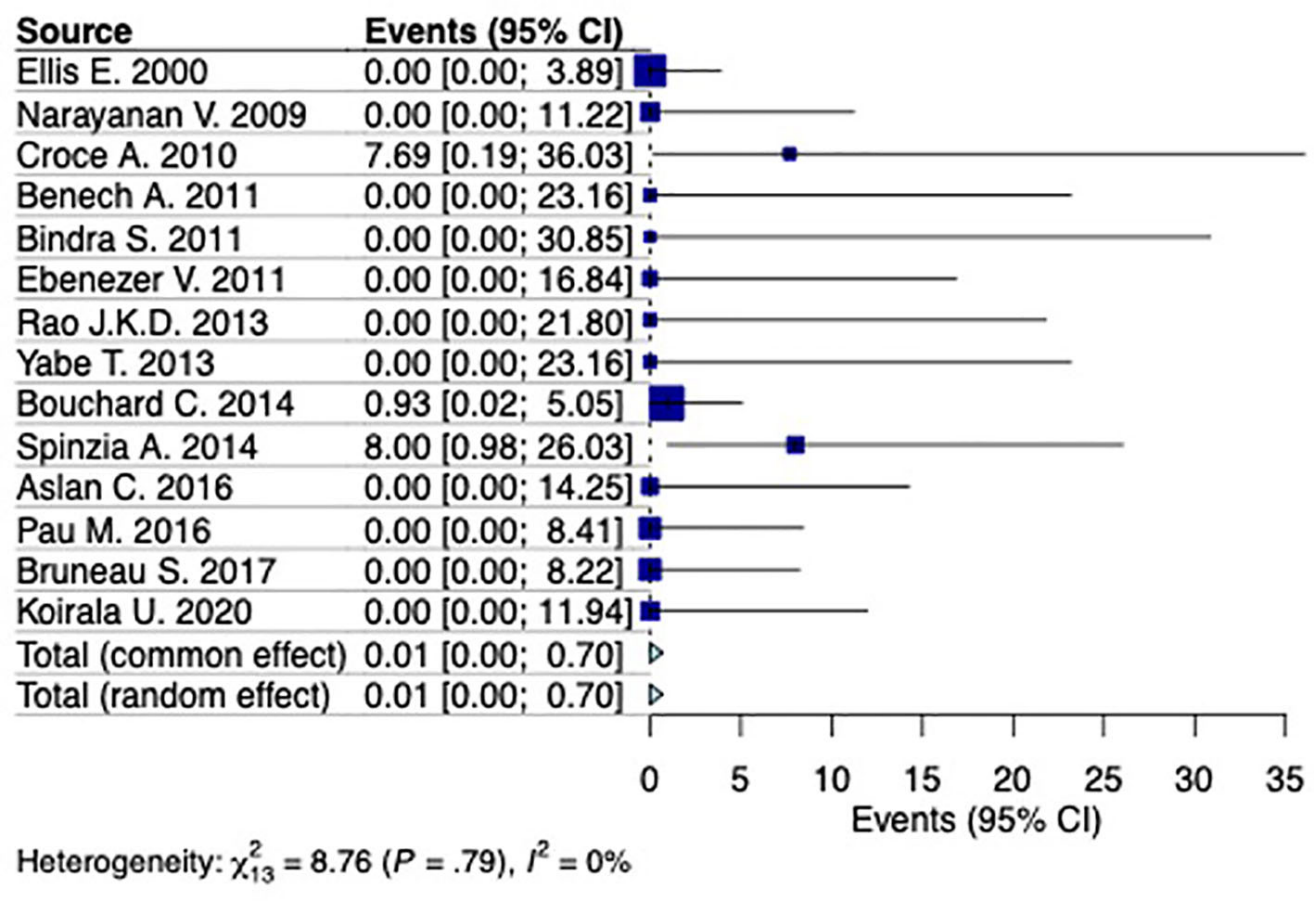
Forest plot evaluating the calculated prevalence of Frey syndrome following extraoral surgical treatment for mandibular fractures.

A random-effects model analysis yielded an initial overall Frey syndrome prevalence following ORIF of 0.01% (95%CI 0%-0.59%) with moderate between studies heterogeneity I ^2^=0% (95%CI 0%-44%, p=0.84). The influence diagnostics and the forest plot illustrating the results of the leave-one-out analysis is presented in Figures 2 and 3. As per them, the study conducted by Zrounba *et a*l. ^38^ identified as influential. After the exclusion of the aforementioned study the estimated prevalence was calculated at 0.01% (95%CI 0%-0.7%) with moderate remaining between-studies heterogeneity I ^2^=0% (95%CI 0%-47%) (p=0.79) ( Figure 4).

### Meta-regression analysis

The meta-regression analysis, which employed continuous variables, unveiled that there exists no statistically significant relationship, whether positive or negative, between the year of publication and the proportion of males. This outcome is visually represented in the Table 2.

**Table 2.** Meta-regression analysis.

Variable	K	Q _M_	Regression coefficient	p-value
Year of publication	14	0.66	0.003 (95%CI -0.004-0.01)	0.42
Proportion of males	12	0.16	-0.001 (95%CI -0.008-0.005)	0.58

K: number of studies.

## Discussion

To the extent of our awareness this study represents the first attempt to evaluate the prevalence of Frey syndrome subsequent to extraoral surgical management of mandibular fractures using a systematic review. Hence, there exists a lack of published data that can be utilized for comparison with our estimation. According to the results of this study, the prevalence was calculated at 0.01% (95%CI 0%-0.7%) with moderate between-studies remaining heterogeneity. The type of surgery and other potential risk factors such as prolonged operative time, patients’ age, gender, fracture pattern, follow-up duration and additional procedures performed may influence the prevalence of Frey syndrome following ORIF for mandibular fractures. Understanding these sources of heterogeneity is crucial for interpreting the prevalence estimate and generalizing the findings to diverse clinical settings. Furthermore, notable heterogeneity is anticipated in the prevalence and incidence estimations owing to the nature of this investigation, stemming from variations in the temporal and geographical contexts of the included studies. As a result, a substantial I ^2^ value within the context of proportional meta-analysis should not automatically indicate data inconsistency. ^27^

The prevalence rate indicates that while Frey syndrome is a relatively uncommon complication, it should still be considered as a potential postoperative outcome following these procedures. Healthcare providers should be aware of the potential risk and actively assess patients for the development of symptoms associated with Frey syndrome during follow-up visits. Early detection and appropriate management strategies can help improve patient outcomes and minimize the impact of this complication on their quality of life. Despite the relatively low prevalence, the impact of Frey syndrome on affected individuals should not be underestimated. Gustatory sweating can cause significant discomfort, embarrassment, and social implications for patients. ^12^^,^^14^ Therefore, it is essential for healthcare providers to educate patients about the potential risks and consequences associated with this complication. Furthermore, appropriate counseling and support should be provided to individuals who develop Frey syndrome to help them cope with the condition effectively. Treatment options for Frey syndrome are focused on symptom management and enhancing patients' quality of life. These options encompass both medical and surgical interventions. Among medical approaches, such as the application of alcohol through topical injection, scopolamine administration, the utilization of glycopyrrolate, and the application of botulinum toxin A, have been proposed and extensively employed for the management of Frey's syndrome. Notably, botulinum toxin A is among the most frequently utilized treatment modalities. ^43^ The collaborative efforts of healthcare professionals from diverse disciplines ensure that individuals with Frey syndrome receive comprehensive and specialized care. This multidisciplinary approach allows for a comprehensive assessment of the condition and enables the development of tailored treatment plans. ^44^

The estimated prevalence rate presented in our study could potentially serve as a valuable benchmark for guiding future research endeavors and informing clinical decision-making processes. Despite the relatively low prevalence, the impact of Frey syndrome on affected individuals should not be underestimated. Given the complexity of this phenomenon, there remains a notable avenue for further investigations aimed at delving into the intricate mechanisms that contribute to the emergence of Frey syndrome subsequent to ORIF procedures for mandibular fractures. Moreover, a distinct need arises for studies that concentrate on formulating preventive strategies and identifying effective management alternatives tailored to patients who are at an elevated risk of encountering Frey syndrome. The outcomes of such endeavors hold the potential to significantly enhance patient well-being and treatment outcomes in a tangible manner.

### Study’s strengths and limitations

The principal strength of the present study is grounded in the methodological approach employed throughout its entirety. However, it is essential to acknowledge the presence of several limitations within this study. Notably, the moderate level of unaccounted heterogeneity present among the included studies does introduce a degree of caution when interpreting the results. As previously noted, the intrinsic nature of this specific study category inherently gives rise to expected outcome variations, thus contributing to the observed moderate heterogeneity. Additionally, irrespective of study nature, potential confounding factors can introduce bias to estimated Frey syndrome prevalence after ORIF for mandibular fractures. These factors ( *e.g.*, extended operative durations, patient age, additional concurrent procedures) underline the need for nuanced consideration of the study's findings.

Furthermore, the inclusion of solely English-language observational studies led to the emergence of reporting bias.

## Data Availability

Figshare: Main characteristics and data outcome of the included studies. https://doi.org/10.6084/m9.figshare.23899944.v1. ^45^ Figshare: PRISMA checklist. https://doi.org/10.6084/m9.figshare.23899986.v1. ^46^ Figshare: PRISMA Flowchart. https://doi.org/10.6084/m9.figshare.24081801.v1. ^47^ Data are available under the terms of the Creative Commons Zero “No rights reserved” data waiver (CC0 1.0 Public domain dedication).
