# Supplementary material for: Prevalence of Frey syndrome following extraoral surgical treatment for mandibular fractures: a systematic review and meta-analysis
Source: F1000Res. 2023 Nov 30;12:1153. Originally published 2023 Sep 14. [Version 2] doi: 10.12688/f1000research.140994.2 (PMC10721962; doi:10.12688/f1000research.140994.2)
Supplement: Peer review history

# Reviewer report: Reviewer response for version 2

**DOI:** 10.5256/f1000research.159391.r227122

**Published:** 2023-12-14

**Authors:** Sadi Memiş

**Affiliations:** 1 Kocaeli University, İzmit, Turkey

Providing information about the fractures in which mandibular region and which extra-oral approaches resulted in Frey Syndrome would have made the study more valuable. However, the authors clearly stated why they could not use these data in the "Study's strengths and limitations" section.

Are the rationale for, and objectives of, the Systematic Review clearly stated?

Yes

Is the statistical analysis and its interpretation appropriate?

I cannot comment. A qualified statistician is required.

Are sufficient details of the methods and analysis provided to allow replication by others?

Yes

Are the conclusions drawn adequately supported by the results presented in the review?

No

Reviewer Expertise:

Oral and Maxillofacial Surgery, Temporomandibular Joint Disorders, Orthognathic Surgery, Oral Implantology, Dentoalveolar Surgery

I confirm that I have read this submission and believe that I have an appropriate level of expertise to confirm that it is of an acceptable scientific standard.

# Reviewer report: Reviewer response for version 2

**DOI:** 10.5256/f1000research.159391.r227121

**Published:** 2023-11-30

**Authors:** Andrea Frosolin

**Affiliations:** 1 University of Siena, Siena, Italy

The article is now suitable for indexing.

Are the rationale for, and objectives of, the Systematic Review clearly stated?

Yes

Is the statistical analysis and its interpretation appropriate?

Yes

Are sufficient details of the methods and analysis provided to allow replication by others?

Yes

Are the conclusions drawn adequately supported by the results presented in the review?

Yes

Reviewer Expertise:

Audiology, phoniatry, maxillofacial clinic and surgery

I confirm that I have read this submission and believe that I have an appropriate level of expertise to confirm that it is of an acceptable scientific standard.

# Reviewer report: Reviewer response for version 1

**DOI:** 10.5256/f1000research.154405.r218204

**Published:** 2023-11-24

**Authors:** Sadi Memiş

**Affiliations:** 1 Kocaeli University, İzmit, Turkey

The study contains some shortcomings and issues that need to be corrected:

1. Under the heading "Abstract"; it is recommended that you share quantitative data in parentheses showing your statistical result (meta-regression value, p value etc.). "In the meta-regression analysis with continuous variables, no statistically significant association was observed."
2. Under the heading "Results and characteristics of the included studies", I recommend that the localizations of mandibular fractures (in which regions they occur) be stated in numbers or percentages.
3. I recommend that the numbers and percentages of extraoral approaches performed under the heading "Prevalence of Frey syndrome following extraoral surgical treatment for mandibular fractures" are given. And I recommend investigating whether there is a statistically significant difference between these extraoral approaches in terms of the development of Frey syndrome. (For example; submandibular approach, retromandibular approach, pre-auricular approach etc.) As it stands now, the data remains dry for the reader to draw conclusions.
4. Under the heading "Discussion"; it should be stated whether definitive information has been obtained from the researched publications that auriculotemporal nerve damage does not occur as a result of trauma in patients who develop Frey syndrome, but occurs after surgery.

I think that the data and discussion of the study without specifying which extraoral approaches were used in the studies and localizing the mandibular fractures do not provide much gain for the readers. I am sorry to say, I do not find it appropriate to index this study in its current form.

Are the rationale for, and objectives of, the Systematic Review clearly stated?

Yes

Is the statistical analysis and its interpretation appropriate?

I cannot comment. A qualified statistician is required.

Are sufficient details of the methods and analysis provided to allow replication by others?

Yes

Are the conclusions drawn adequately supported by the results presented in the review?

No

Reviewer Expertise:

Oral and Maxillofacial Surgery, Temporomandibular Joint Disorders, Orthognathic Surgery, Oral Implantology, Dentoalveolar Surgery

I confirm that I have read this submission and believe that I have an appropriate level of expertise to state that I do not consider it to be of an acceptable scientific standard, for reasons outlined above.

# Response

**Published:** 2023-11-25

**Authors:** Evangelos Kostares

**Affiliations:** Microbiology, National and Kapodistrian University of Athens, Athens, Attica, Greece

On behalf of all authors, we would like to thank you for your kind words and valuable input regarding our manuscript.  Please find a point-to-point response to each of your comments.

**Comment #1**

 Under the heading "Abstract"; it is recommended that you share quantitative data in parentheses showing your statistical result (meta-regression value, p value etc.). "In the meta-regression analysis with continuous variables, no statistically significant association was observed."

**Response #1**

 Thank you for your valuable feedback. While we understand the importance of providing quantitative data in the abstract, the meta-regression analysis did not yield statistically significant results. Given the absence of statistical significance, we believe the clarity of our statement, "In the meta-regression analysis with continuous variables, no statistically significant association was observed," appropriately communicates the outcome without introducing unnecessary numerical information. For a comprehensive understanding, readers can refer to Table 2, where the meta-regression analysis results are detailed.

**Comment #2**

 Under the heading "Results and characteristics of the included studies", I recommend that the localizations of mandibular fractures (in which regions they occur) be stated in numbers or percentages.

**Response #2**

 Thank you for your valuable feedback. We recognize the significance of presenting mandibular fracture localizations through numerical data or percentages. It's essential to emphasize that our study functions as a meta-analysis of observational studies, involving a diverse patient population with varying fracture types. Although we contemplated conducting a subgroup analysis based on regional fracture occurrences, practical challenges arose due to the diverse range of fractures observed within each study (the absence of information concerning the classification of fractures in every study renders the implementation of this suggestion impossible). In an effort to uphold the overall integrity and statistical robustness of our study, we chose a more comprehensive approach, considering the available data. The decision not to exclude these studies was made to prevent compromising the reliability and statistical power of our research. The inherent heterogeneity among the included studies, in terms of both design and reporting standards, presents challenges that make it difficult, if not impossible, to conduct a precise analysis of fracture localizations. Introducing specific numbers or percentages in this context could potentially result in misleading or inaccurate conclusions. Despite these challenges, we have taken your feedback into account and addressed it in the revised version of the manuscript. Specifically, we have incorporated a clear acknowledgment of these challenges in the "Study’s strengths and limitations" section of the revised study. We believe that by openly recognizing this limitation, readers will gain a comprehensive understanding of the constraints associated with our methodology. Once again, we appreciate your constructive feedback, which has undoubtedly contributed to the refinement of our manuscript.

**Comment #3**

 I recommend that the numbers and percentages of extraoral approaches performed under the heading "Prevalence of Frey syndrome following extraoral surgical treatment for mandibular fractures" are given. And I recommend investigating whether there is a statistically significant difference between these extraoral approaches in terms of the development of Frey syndrome. (For example; submandibular approach, retromandibular approach, pre-auricular approach etc.) As it stands now, the data remains dry for the reader to draw conclusions.

**Response #3**

 We express gratitude for your insightful suggestion concerning the exploration of numbers and percentages related to extraoral approaches in the context of "Prevalence of Frey syndrome following extraoral surgical treatment for mandibular fractures". While we acknowledge the significance of investigating potential variations in the development of Frey syndrome among different extraoral approaches (such as submandibular, retromandibular, pre-auricular, etc.), we encountered practical challenges during the analysis. Initially, we contemplated performing a subgroup analysis focused on extraoral procedures; however, the diverse array of techniques utilized in each study rendered it impractical. To maintain the overall integrity and statistical robustness of the study, we opted for a broader scope with the available data. Excluding these studies could have compromised the reliability and statistical power of the study. Meta-analyses inherently rely on synthesizing data from diverse sources, and the lack of standardized reporting across primary studies is a common challenge. Attempting to include such details without uniformity across the studies could introduce inaccuracies and potentially mislead readers. In light of these challenges, we find ourselves unable to perform the suggested analysis. We believe that presenting incomplete or disparate data might compromise the integrity of our findings, leading to inaccurate interpretations and conclusions. Our study aimed to synthesize the available literature comprehensively, and the limitations in reporting within the primary studies constrain the depth of our analysis. Despite these challenges, we have taken your feedback into account and addressed it in the revised version of the manuscript. Specifically, we have incorporated a clear acknowledgment of these challenges in the "Study’s strengths and limitations" section of the revised study. We believe that by openly recognizing this limitation, readers will gain a comprehensive understanding of the constraints associated with our methodology. Once again, we appreciate your constructive feedback, which has undoubtedly contributed to the refinement of our manuscript.

**Comment #4**

 Under the heading "Discussion"; it should be stated whether definitive information has been obtained from the researched publications that auriculotemporal nerve damage does not occur as a result of trauma in patients who develop Frey syndrome, but occurs after surgery.

 I think that the data and discussion of the study without specifying which extraoral approaches were used in the studies and localizing the mandibular fractures do not provide much gain for the readers. I am sorry to say, I do not find it appropriate to index this study in its current form.

**Response #4**

 Thank you for your valuable feedback. In response to your suggestion regarding definitive information on auriculotemporal nerve damage, we acknowledge the importance of this aspect. However, after a thorough review of the included studies, we found a lack of conclusive evidence on whether auriculotemporal nerve damage is solely associated with surgical interventions or if trauma alone can lead to such damage in Frey syndrome patients. As the existing literature does not provide a definitive answer to this question, we refrained from making statements that could potentially mislead the readers. We also acknowledge your concern about the study's perceived lack of gain for readers due to the absence of specific details on extraoral approaches and localized mandibular fractures. Regrettably, the studies included did not consistently offer the desired level of detail. Attempting to force inclusion of specific information might compromise the integrity and accuracy of the meta-analysis. It is essential to balance the desire for comprehensive reporting with the need to accurately reflect the available evidence. Our study is designed to function both as a rudimentary assessment and as a groundwork for subsequent prospective and retrospective investigations. In light of this limitation, we have taken your feedback into account and addressed it in the revised version of the manuscript. Specifically, we have incorporated a clear acknowledgment of these challenges in the "Study’s strengths and limitations" section of the revised study. We believe that by openly recognizing this limitation, readers will gain a comprehensive understanding of the constraints associated with our methodology. Once again, we appreciate your constructive feedback, which has undoubtedly contributed to the refinement of our manuscript.

# Reviewer report: Reviewer response for version 1

**DOI:** 10.5256/f1000research.154405.r211992

**Published:** 2023-10-13

**Authors:** Andrea Frosolin

**Affiliations:** 1 University of Siena, Siena, Italy

I read with interest your SR-MA about mandibular fractures and the prevalence of Frey syndrome following open reduction and internal fixation (ORIF).

 Just some minor revisions:

- Consider rephrasing this sentence for clarity: "Despite being widely esteemed as an efficacious and secure procedure, open reduction and internal fixation (ORIF) introduces significant intricacies..."
- When discussing surgical techniques, consider mentioning specific techniques or advancements that have been proposed or used in preventing Frey syndrome (e.g. Gennaro *et a*l., 2013 ^1^). This adds depth to the discussion.
- There are specific pharmacological interventions that have shown promise in preventing or managing Frey syndrome, consider mentioning them (e.g. Tugnoli *et al., *2002 ^2^).

These are minor suggestions for improvement, and the text is generally well-written and informative.

Are the rationale for, and objectives of, the Systematic Review clearly stated?

Yes

Is the statistical analysis and its interpretation appropriate?

Yes

Are sufficient details of the methods and analysis provided to allow replication by others?

Yes

Are the conclusions drawn adequately supported by the results presented in the review?

Yes

Reviewer Expertise:

Audiology, phoniatry, maxillofacial clinic and surgery

I confirm that I have read this submission and believe that I have an appropriate level of expertise to confirm that it is of an acceptable scientific standard.

## References

[1] : Use of irradiate animal pericardium membrane for prevention of Frey's syndrome after parotidectomy. *Eur Rev Med Pharmacol Sci* .2013;17(4) :548-51 23467957

[2] : The role of gustatory flushing in Frey's syndrome and its treatment with botulinum toxin type A. *Clin Auton Res* .2002;12(3) : 10.1007/s10286-002-0026-x 174-8 10.1007/s10286-002-0026-x 12269549
